# Hydrogen and helium trapping in hcp beryllium

**DOI:** 10.1038/s42004-023-00877-7

**Published:** 2023-04-21

**Authors:** Nikolai Zimber, Judith Lammer, Pavel Vladimirov, Gerald Kothleitner, Vicki J. Keast, Michael Dürrschnabel, Michael Klimenkov

**Affiliations:** 1 Karlsruhe Institute of Technology (KIT), Institute for Applied Materials – Applied Materials Physics (IAM-AWP), Eggenstein-Leopoldshafen, Germany; 2 Institute of Electron Microscopy and Nanoanalysis (FELMI), Graz University of Technology & Graz Centre for Electron Microscopy (ZFE), Graz, Austria; 3 School of Information and Physical Sciences, University of Newcastle, Callaghan, NSW Australia

**Keywords:** Transmission electron microscopy, Nuclear fusion and fission, Chemical hydrogen storage

## Abstract

Even though hydrogen-metal surface interactions play an important role in energy technologies and metal corrosion, a thorough understanding of these interactions at the nanoscale remains elusive due to obstructive detection limits in instrumentation and the volatility of pure hydrogen. In the present paper we use analytical spectroscopy in TEM to show that hydrogen adsorbs directly at the (0001) surfaces of hexagonal helium bubbles within neutron irradiated beryllium. In addition to hydrogen, we also found Al, Si and Mg at the beryllium-bubble interfaces. The strong attraction of these elements to (0001) surfaces is underlined with ab-initio calculations. In situ TEM heating experiments reveal that hydrogen can desorb from the bubble walls at *T* ≥ 400 °C if the helium content is reduced by opening the bubbles. Based on our results we suggest the formation of a complex hydride consisting of up to five elements with a remarkably high decomposition temperature. These results therefore promise novel insights into metal-hydrogen interaction behavior and are invaluable for the safety of future fusion power plants.

## Abstract (web summary)

Hydrogen–metal surface interactions play an important role in energy technologies and metal corrosion, but a thorough understanding of these interactions at the nanoscale remains elusive due to detection limits and the volatility of pure hydrogen. Here, the authors use transmission electron microscopy to show that hydrogen adsorbs directly at the (0001) surfaces of hexagonal helium bubbles within neutron irradiated beryllium.

## Introduction

Hydrogen is seen as one of the key elements in Europe’s pathway for the clean energy transition^1^. For the envisaged sector coupling hydrogen is considered as the only coupling element capable to link electricity and gas^2^. Furthermore, it is planned to establish a hydrogen economy in which mainly or exclusively hydrogen is used as an energy carrier^1,2^. In order to implement these technological challenges, a profound understanding of the interaction behavior of hydrogen with materials is required. In particular, the interplay between hydrogen isotopes and metal surfaces is of special interest. As an undesired impurity, hydrogen can lead to a deterioration of the mechanical properties in many materials including steels^3^, aluminum^4^ and titanium^5^. On the other hand, hydrogen storage materials are being developed to store energy with a similar energy density as compared to fossil fuels^6–8^. In addition, the metal-hydrogen interaction behavior plays an important role in the development of materials for nuclear fusion reactors^9^.

In future fusion power plants large amounts of helium (^4^He) and tritium (^3^H) will be generated by neutron induced transmutation within the structural and functional components of the reactor^9^. As it is well known, these two transmutation products form gas bubbles together with vacancies, whereby their diameters can be up to 200 nm^9–11^.

Beryllium is considered as a plasma-facing material for the international thermonuclear experimental reactor (ITER), the world’s largest fusion experiment, but also as an effective neutron multiplier material for the ITER Test Blanket Module (TBM) as well as for the first fusion demonstration power plant DEMO^12–14^. In order to evaluate the irradiation resistance of beryllium, which is in particular important for its use as neutron multiplier, various long-term neutron irradiation campaigns have been performed in the past. Evaluation of the latest campaign, HIDOBE-02^15^, revealed a strong trapping of the β-radioactive tritium (*t*_1/2_ ≈ 12.3 y) within the material. Even at elevated irradiation temperatures of up to 650 °C more than 30 % of the theoretically evaluated tritium content was retained during the irradiation^16^ which represents a significant safety risk since these temporary trapped quantities could be released in an uncontrolled manner in the event of an accident. Therefore, it is essential to gain an in-depth understanding of where and, more importantly, why tritium is trapped within the material. It is generally assumed that both, helium and hydrogen are located directly inside bubbles since both elements are released simultaneously when bulk samples are heated to temperatures ≥ 1100 °C after irradiation^17–20^.

While the interplay between beryllium and hydrogen isotopes was studied already extensively in computer simulations^21–26^ there is still a lack of experimental data. Mapping hydrogen locally is usually challenging and was performed in the past particularly by atom probe tomography (APT)^27–29^. The large quantities of hydrogen within irradiated materials pave the way to analyze metal-hydrogen interaction at the nanoscale with different analysis methods. Electron energy loss spectroscopy (EELS) measurements revealed the co-existence of helium and tritium in hexagonal prismatic bubbles within beryllium^30^. While the noble gas helium resides directly inside the bubble and is homogenously distributed over the entire volume, the hydrogen isotope predominantly resides at the basal planes of the bubbles. Although these findings proof the existence of the two elements inside bubbles, they could not elucidate the underlying mechanisms of the observed strong hydrogen trapping within the material.

This study addresses the metal-hydrogen interaction within helium-bubbles in neutron irradiated beryllium and provides evidence of strongly trapped hydrogen within closed helium bubbles. Our results reveal that the presence of He stabilizes the hydride within the bubbles and that additional impurities, promoting complex hydrides, force the decomposition temperatures to unexpected high values.

## Experimental results and discussion

### Measurements of closed gas bubbles

**Fig. 1:**
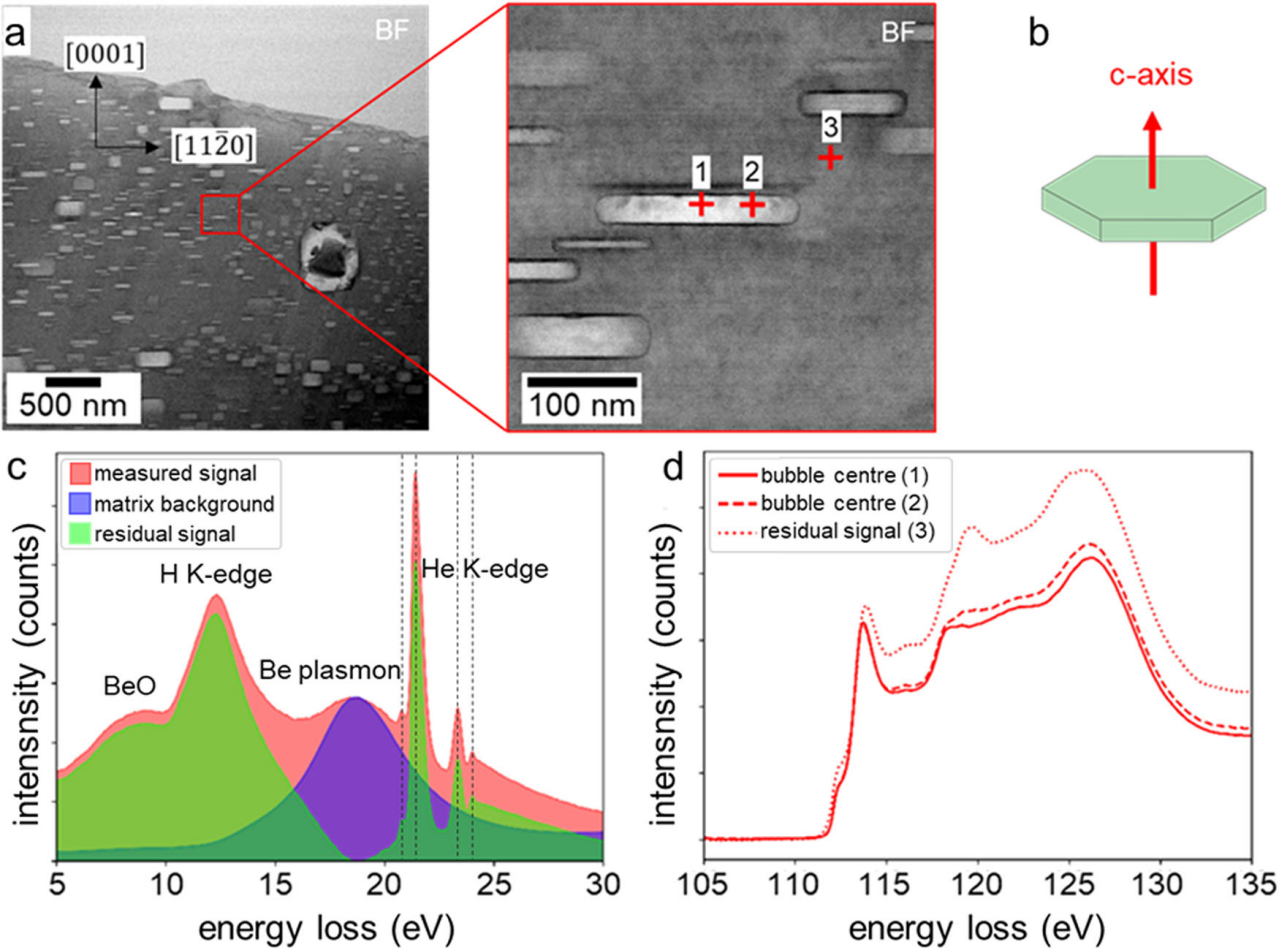
EELS measurements of closed hexagonal helium bubbles within neutron irradiated beryllium. **a** Bright-field STEM image of the irradiated Be microstructure (left) and close up of an investigated bubble (right). Cross (1) marks the position of the spectrum shown in **c**, cross (2) the position used to open the bubble with the electron beam described in Fig. 3 and the position at cross (3) served for matrix background acquisition. **b** Scheme of a hexagonal prismatic disc shape. **c** Low-loss region of an EEL spectrum acquired in the bubble center after matrix background subtraction (green residual signal). The dominate peak at 12.9 eV is associated to hydrogen. The four spikes (marked with dashed lines) appearing in the 22 eV region correspond to the He K-edge. **d** Variation in the Be K-edge as a function of the relative position within the closed gas bubble as it is indicated by the numbers in 1 (**a**, right)^68^.

The hexagonal symmetry of the beryllium matrix implies a peculiar form of the gas bubbles being hexagonal prisms. The bright-field scanning transmission electron microscopy (STEM) image in Fig. 1a shows the typical microstructure of beryllium after neutron irradiation at 600 °C distinguished by a high number density of helium bubbles visible as light rectangles. They are observable as hexagons if viewed along the c-axis in (0001) direction or as rectangles along the basal planes perpendicular to the (0001) direction (see Fig. 1a, b).

A typical EELS low-loss spectrum (presented in Fig. 1c) from the interior of a bubble—with a bubble diameter of 150 nm parallel to electron beam within a 210 nm thick FIB lamella—clearly depicts several signals: At 18.4 eV the beryllium bulk plasmon emerges. Left of the bulk plasmon a signal at 12.9 eV appears which is associated to hydrogen^31,32^. The He K-edge is visible around 22 eV. Note that, to our knowledge, there are four different transition levels of the He-atom visible for the first time in a conventional EELS spectrum corresponding to the following transitions: 1s2→1s2s (20.7 eV), 1s2→1s2p (21.47 eV), 1s2→1s3p (23.36 eV) and 1s2→1s4p (23.99 eV). The additional plasmon with a maximum at 8.5 eV correlates with the formation of BeO that forms on the surface of the FIB lamellae as a thin oxide layer but does not interact with the bubbles (see Supplementary Fig. 2).

**Fig. 2:**
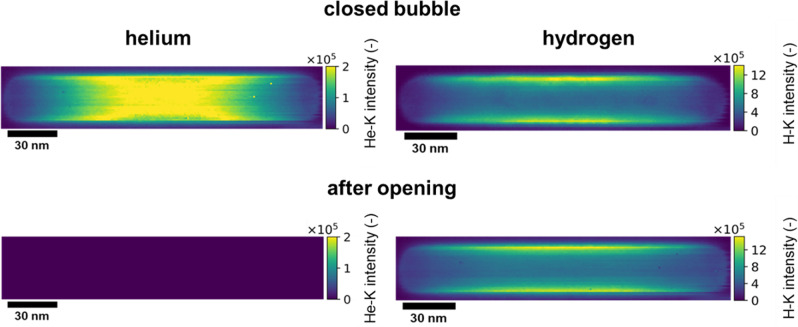
Helium and hydrogen distribution within hexagonal bubbles in neutron irradiated beryllium. Helium and hydrogen intensity-maps before and after opening the bubble in situ in the microscope using the electron beam^68^.

When depicting the hydrogen and helium distribution within the bubble (Fig. 2a), it can be clearly seen that hydrogen predominantly occupies the basal planes of the bubbles while the integrated He intensity follows the shape of the hexagonal prismatic bubble. A closer look at the He-intensity map reveals the absence of helium in the immediate vicinity of the basal planes of the bubbles, i.e., at the Be-bubble interface. While the low-loss spectrum does not change qualitatively across the bubble, there are major changes in the high-loss signal. As it can be seen from Fig. 1d) the shape of the Be K-edge changes depending on the position where it was acquired. These modifications reflect the influence of atoms in the direct vicinity of Be and might indicate a change in the binding character.

### Opening the bubbles

**Fig. 3:**
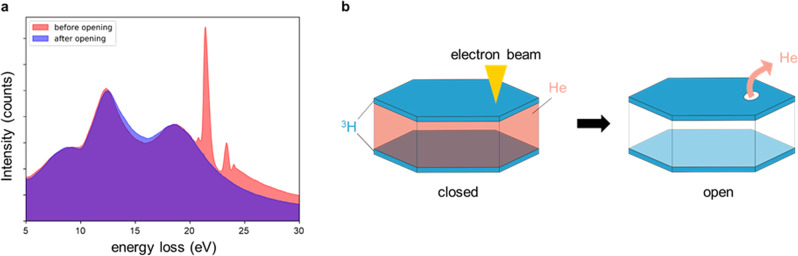
In situ opening of bubbles with the electron beam. **a** Comparison of the EEL spectra, before (red) and after (blue) the bubble was opened. **b** Schematic of the proposed He/H arrangement within the hexagonal bubbles^68^.

In order to verify whether hydrogen is just loosely adsorbed at the bubble surface or strongly bound within the bubble, we opened the bubbles by burning a hole in the bubble walls using the electron beam of transmission electron microscopy (TEM). The intention was to see, whether hydrogen remains inside the bubble or escapes. This procedure is illustrated in Fig. 3b. From Fig. 3a it can be directly seen that the helium signal completely vanished as a consequence of the bubble opening. The hydrogen intensity, however, remained nearly the same. The integrated He and H intensities after the bubble opening are depicted in Fig. 2b.

### In situ heating of bubbles

**Fig. 4:**
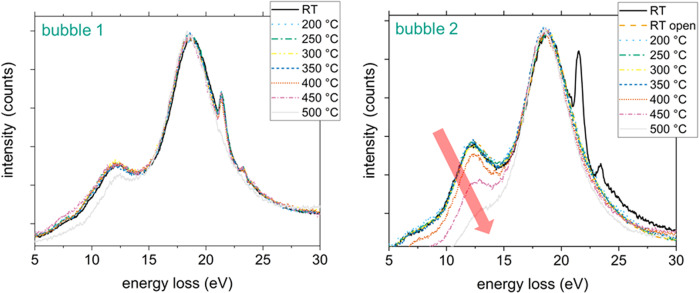
In situ EELS measurements of bubbles. EEL spectra obtained from a closed (bubble 1) and an opened bubble (bubble 2) at room temperature and during stepwise heating. While the hydrogen peak in the closed bubble does not show a significant change during heating, the hydrogen signal vanishes with increasing temperature for the opened bubble.

As a next step we performed in situ heating experiments with closed and opened bubbles to analyze the hydrogen behavior during stepwise heating. Figure 4 shows the EEL spectra from bubble surfaces at room temperature and during a stepwise temperature increase of two bubbles located next to each other (see Supplementary Fig. 1). Bubble 1 was kept closed as reference bubble but got opened unintentionally at 450 °C. Bubble 2 was intentionally opened with the electron beam at room temperature to allow helium to escape the bubble before the heating procedure. As indicated by the EEL spectra of bubble 1 (see Fig. 4) the hydrogen-peak intensity remains more or less constant even at elevated temperatures as long as the bubble is closed. However, when opening the bubble prior to heating, the hydrogen signal clearly vanished continuously from 400 °C on (see bubble 2 in Fig. 4). Comparing the EELS signals from bubble 1 and bubble 2 at 500 °C reveals that the hydrogen signal decreases stronger for bubble 2 which is most likely caused by the fact that both bubbles are located at different depths within the lamella. Therefore, the energy impact from the electron beam should be different. Moreover, bubble 2 seems to have higher hydrogen and helium contents compared to bubble 2 since the peak height ratio hydrogen/beryllium and helium/beryllium is larger for bubble 2. Therefore, it can be expected that bubble 2 can loose more hydrogen than bubble 1 which results in a stronger decrease of the hydrogen peak.

### He gas-pressure

The noble atomic gas helium distributes itself homogeneously within the closed gas bubble with a density of ~10–12 at/nm^3^ (53–64 % of the density of liquid helium at its boiling point and atmospheric pressure^33^), which theoretically results in a pressure build up of up to 4.4 × 10^−2^ GPa in the bubble interior (see Supplementary Methods for calculation details). It should be noted that for such densities both helium and hydrogen cannot be considered as ideal gases and are strongly interacting with each other.

### Chemical bonding of H at the bubble walls

Hydrogen predominantly occupies the basal planes of the bubble and appears to be strongly bound there, also when the bubble is opened at room temperature. Furthermore, hydrogen is strongly bound to the basal planes even at elevated temperatures if the bubble remains closed (see Fig. 4a). We were able to measure closed bubbles filled with hydrogen and helium up to 750 °C (see Supplementary Fig. 3). Beyond this temperature our Be-lamellae were no longer thermally stable. If the bubble is opened before heating, hydrogen starts to desorb from the basal planes from 400 °C (673 K) on and seems to be completely vanished at 500 °C (773 K). This temperature is significantly higher than temperatures around 420 K (147 °C) associated with the hydrogen-isotope desorption peak from the external beryllium surface as observed both after deuterium implantation^34^ or thermal loading with tritium^35^. Implantation experiments like the ones performed in the aforementioned publication, but also in other investigations^36,37^ are often performed with BeO covered surfaces since beryllium oxidizes quickly in air. Experiments on atomic deuterium exposure of oxygen covered external beryllium surfaces by Lossev and Küppers^38^ have revealed two major D_2_ desorption peaks at 320 K and 450 K ascribed to the desorption from oxygen covered and clean beryllium surfaces, respectively. At low D-exposures another peak at 510 K can be resolved in addition. In our experiment helium bubbles were grown inside bulk beryllium during neutron irradiation and opened by the electron beam under vacuum. The internal surface of the bubble was thus not exposed to oxygen. Although some oxygen traces can be present in vacuum, it is not expected to have any notable oxygen coverage inside just opened bubbles also taking into account a small size of the opening hole. Therefore, only two H-release peaks, namely, at 450 K (177 °C) and 510 K (237 °C) were initially expected: However, both temperatures are notably below the onset of tritium desorption in our experiments at 673 K (400 °C). It should be also noted that no difference in surface desorption of hydrogen and deuterium were found in ref. ^38^. Moreover, the isotope effects commonly result in a change in activation energy of about 0.1 eV and cannot be responsible for such large temperature difference (223 °C).

In general, one must be cautious when comparing results from implantation experiments to our neutron irradiated samples. In the case of exposure or implantation experiments, atomic hydrogen is entering material through the external surface. It is chemisorbed at the surface first and should overcome a barrier of up to 3 eV for beryllium to be dissolved in the bulk material^26^. Therefore, in surface exposure experiments the main part of deuterium is trapped by the material surface. Neutron irradiation leads to the formation of vacancies (V) that strongly trap hydrogen with a binding energy of up to 1.3 eV^23^. Within helium bubbles the binding energy is even 1.8–2.0 eV^10^. The He-V binding energy itself can reach more than 3 eV^22^. From these binding energies it can be concluded, that both helium and tritium are strongly trapped within bubbles inside of neutron irradiated beryllium.

**Fig. 5:**
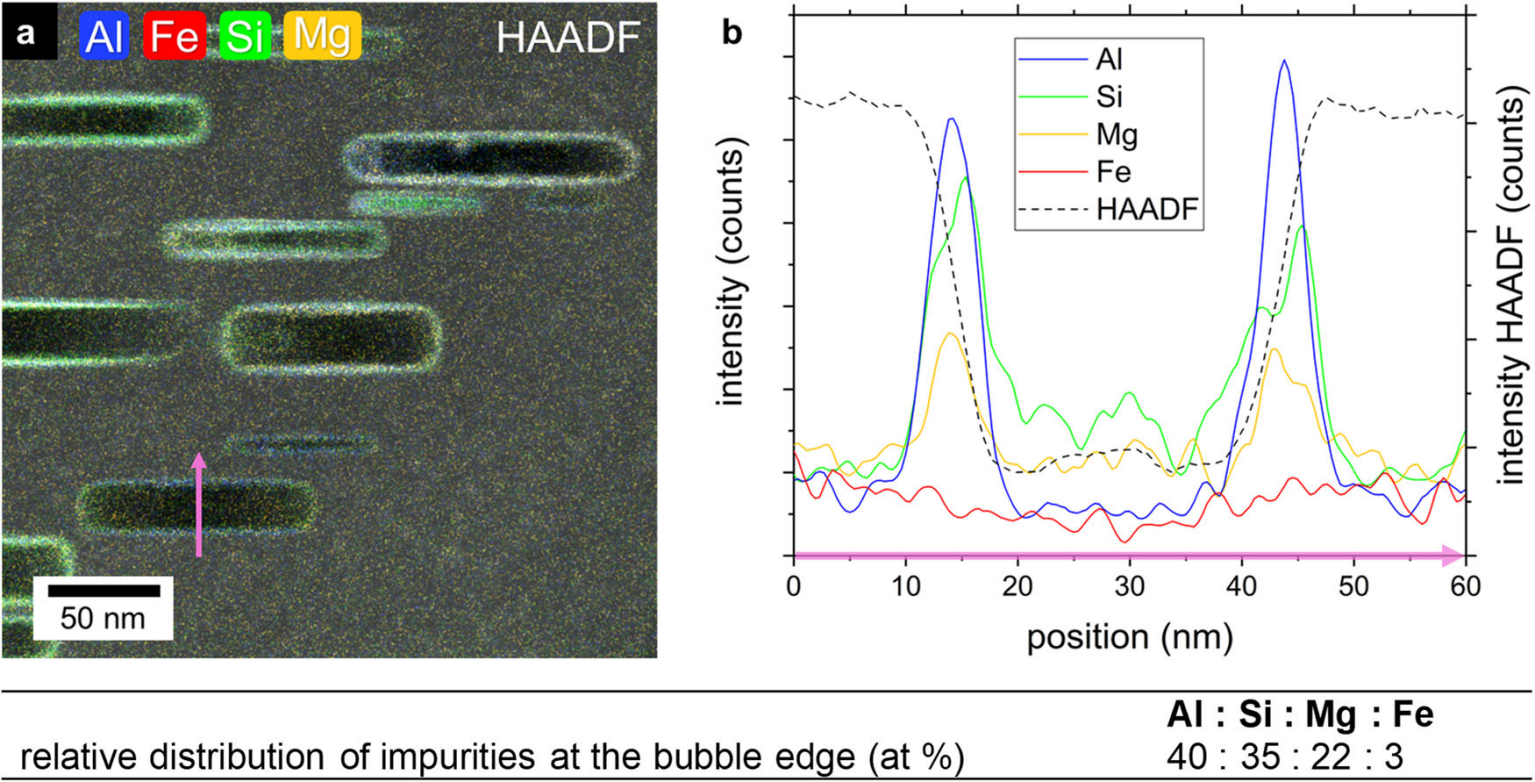
Accumulation of impurities at bubble walls. **a** EDX scan of bubbles in beryllium with overlaid high-angular annular dark-field (HAADF). The elements Al, Mg and Si can be found at the bubble surface. The concentration profile of the major impurity, iron, is plotted for comparison. The purple arrow marks the extraction position for the concentration profiles shown in **b**^68^.

**Fig. 6:**
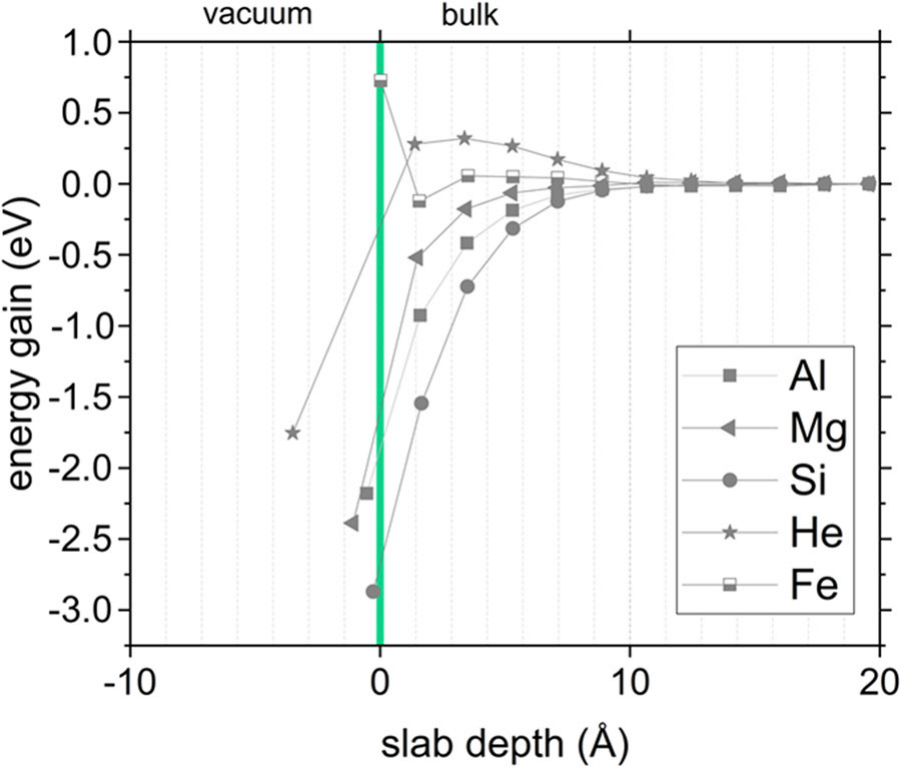
DFT calculations revealing interaction of various impurities with (0001) beryllium surfaces. Energy gain due to substitutional impurities in a Be slab as a function of the distance from the slab surface for Al, Mg, Si, He and Fe. The substitutional helium is slightly repelled by the surface, but receives a significant energy gain by jumping into the bubble. Only the presence of iron at the surface is energetically unfavorable, while all other elements lead to a significant energy reduction.

Density functional theory calculations (DFT)^39^ as well as thermal desorption spectrometry experiments^40^ suggest the formation of BeH_2_ on the surface of hydrogen saturated bulk beryllium. According to the simulations in ref. ^39^ the continuous segregation of hydrogen on (0001) Be surfaces can lead to a substantial surface reconstruction and a subsequent formation of irregular BeH_2_-chains at the surface. Indeed, the Be K-edge shape changes in the bubble interior compared to the bulk indicating a change in the binding character, which could be attributed to the formation of BeH_2_. However, the observed H-desorption temperatures, starting at 400 °C are higher than the values reported for the decomposition temperature of BeH_2_ (~300 °C^40,41^). Furthermore, we were able to detect accumulation of impurities such as Al, Si and Mg at the inner bubble walls (see Fig. 5) using energy-dispersive X-ray spectroscopy (EDX). These elements have a strong binding energy of up to 1.0 eV with vacancies^10^. Our ab initio calculations show that the preferential position of these elements is inside the bubbles, directly at the bubble-matrix interface (see Fig. 6). As a consequence, they form halos around bubbles as can be seen in Fig. 5. It is not very likely that these solutes trap the hydrogen on their own as the decomposition temperatures of MgH and AlH_3_ are reported to be < 300 °C^42,43^ and Si_x_H_x_ compounds on the other hand are usually either gaseous or liquid depending on the Si/H ratio. Therefore, it seems to be most likely that a hydride compound, possibly consisting of 5 elements (Be, Al, Si, Mg, H) with a higher decomposition temperature than hydrides consisting of single metal element, was formed at the basal planes. DFT calculations of hydrogen behavior on Al^44^ and Mg^45^ surfaces have shown in the past that even small amounts of impurity atoms are sufficient to significantly influence hydride formation. The presence of the impurity atoms changes the electronic structure on the surface and thus the interaction of hydrogen with the metal surfaces. In closed bubbles the hydride-compound is apparently stabilized by the high helium density in the bubble interior, even at temperatures of up to 750 °C. Moreover, due to the relatively high helium density, the atomic shells of neighboring helium atoms are overlapping which results in a repulsive interaction between their electrons which is measurable as a blue shift of the He K-edge (see Supplementary Fig. 4) in the EELS spectra^46,47^, in our case up to 0.3 eV. It might be concluded that in closed bubbles, the high helium density prevents the complex hydride from decomposing and consequently hydrogen from desorbing from the bubble surfaces due to the limited space inside the bubbles. Under such conditions, it is impossible for hydrogen to form a molecule which is a necessary condition for desorption. Diffusion back into the bulk is also hindered because of the associated barrier of almost 3 eV^26^.

### Summary and conclusion

We shed light on the metal-hydrogen interaction within nanoscaled bubbles in neutron irradiated beryllium using a combination of EDX and STEM (in situ) EELS. In particular we have shown with nanoscaled elemental maps that hydrogen is strongly bound to the inner (0001) beryllium surfaces, even at elevated temperatures as long as the bubbles remain closed. The hydrogen EELS signal vanished only from bubbles that were opened prior or during the heating procedure. Consecutive hydrogen desorption took place at temperatures ≥400 °C, which is approximately 100 °C above the expected decomposition temperature of pure BeH_2_^40,41^ and also above most known metal hydrides^48^. Our results demonstrate that hydrogen inside the bubbles is not completely in a gaseous state, but the majority of hydrogen atoms is bound to the inner bubble walls, presumably in the form of a complex-hydride including ^3^H, Be and the additional impurities Al, Mg, Si. Such hydrides seem to be stabilized above their normal decomposition temperature by the large amounts of helium in the bubble interior. These findings can be used to explain the high tritium release temperature of T ≥ 1100 °C in TPD experiments^17–19^. The necessity of heating the pebbles to such high temperatures raises serious problems that have not yet been solved, among others because the surrounding structural materials are not designed for these temperatures^49^.

Deeply understanding the interaction behavior of hydrogen with metal surfaces—and as a consequence the tritium retention in fusion materials—is crucial for the safety of future fusion reactors. Moreover, our results are not only exclusively relevant for this technology. Our findings on trapping mechanisms and increased desorption temperatures help to deepen the fundamental understanding of the metal-hydrogen interaction behavior relevant for metal-hydrogen systems used in aerospace applications, fuel cells or metallurgical applications^50^. And—more importantly at the moment—they are significant for the energy transition, since hydrogen is seen as a central element of the planned sector coupling in Europe^1,2^. Hydrogen-interface effects with metals play a critical role in the production, storage and energetic use of hydrogen and are therefore a central component in the technological implementation of the desired hydrogen economy^51,52^. Nanoscaled mapping of hydrogen can help understanding the atomic origin of phenomena like hydrogen assisted embrittlement and cracking of structural materials^53^ or hydrogen storage and release mechanisms^54,55^. Our results can therefore help to achieve a fundamental change in energy supply for a post-fossil economy.

## Methods

### Fabrication and irradiation of the Be samples

A total of 1 mm large Be-pebbles as they will be used in later reactors, were produced by rotation electrode process (REP) by NGK Insulators Ltd., Japan^56^. The pebbles were then gently pressed into a containment (so called constrained pebbles) and irradiated for 1274 full power days (FPDs) in 47 reactor cycles within four-years irradiation within the framework of the high dose beryllium neutron irradiation programme (HIDOBE-02) performed in the material testing High Flux Reactor (HFR) in Petten, the Netherlands^15^. Due to the experimental setup, the damage as well the gas production rate increases with neutron flux followed by the irradiation temperature as it can be seen from Table 1. For the present study we only took samples from the highest irradiation temperature since the high helium and tritium contents allowed for a detailed investigation. The unit dpa (displacements per atom) indicates how often each lattice atom has been ballistically displaced from its lattice position during the irradiation period. For more details on neutron irradiation damage see^9^. Since temperatures in neutron irradiation experiments vary over time temperatures in Table 1 are given as target, average and maximum. During irradiation temperatures were controlled with 24 thermocouples within the sample holder. If necessary, the temperature was adjusted by means of a tailored gas gap between an outer stainless steel sampler holder tube and the containment tube. For further information on irradiation conditions, please note the articles by Hegeman et al.^15^. and Klimenkov et al.^10^.

**Table 1:** Overview of the irradiation parameters for the constrained Be pebbles that were irradiated during the high dose beryllium neutron irradiation programme HIDOBE-02 in 1274 full power days.

Irrad. temperature (°C)			Fluence (10^25^ m^−2^)		Dpa	He (appm)	^3^H (appm)
Target	Average	Max.	Therm.	Fast >1 MeV			
405	387	425	7.89	1.6	21	3632	367
500	487	523	9.87	14.3	29	4751	502
620	600	645	11.3	16.8	34	5524	596

For this study we only investigated the high temperature samples, irradiated at 600 °C as they have the highest helium and hydrogen content. The chemical composition of the pebbles is given in Table 2.

**Table 2:** Chemical composition of the irradiated 1 mm Be pebbles^68^.

Element	Concentration (wppm)
Fe	1050
Al	376
Ni	153
Cu	113
Mg	116
Mn	104
Cr	102
Si	305
U	103
Ti	42
Zr	31
Au	20
V	10
O	153
C	239
N	<20
Be	balance

### Sample preparation

We embedded the irradiated pebbles into a conductive epoxy resin and mechanically polished them. The obtained cross-sections were then examined using a polarized light microscope to determine the regions of interest. Afterwards we prepared thin lamellae from the chosen areas using a FEI Helios Dual Beam microscope. For the EELS investigations we thinned the samples to a thickness of 40–210 nm. For the heating experiments the lamellae were mounted on nanochips produced by DENSsolutions.

### Transmission electron microscopy

We studied the lamellae using a probe aberration-corrected scanning transmission electron microscope (FEI Titan^3^G2 60–300) equipped with a high-brightness electron gun (x-FEG) and an electron monochromator. High-angular annular dark-filed (HAADF) and bright-field (BF) detectors were used to acquire the STEM micrographs. All TEM measurements were performed under high-vacuum conditions.

For the EELS measurements we operated the microscope at 300 kV using a Dual EELS–Gatan Imaging Filter (GIF) Quantum as well as a direct-electron-detection camera K2. The obtained energy resolution was between 0.12 and 0.2 eV with convergence and collection angles of 15.89 mrad and 18.77 mrad, respectively. The dispersion was set to 0.025 eV/channel and the current was approximately 125 pA.

In order to detect as much helium and hydrogen signal as possible, we oriented the samples relative to the c-axis of the hexagonal lattice of the material such that the (0001) planes of the hexagonal prismatic bubbles are perpendicular to the optical axis (see Fig. 1b).

Spectrum images (SI) for several bubbles at different positions in the material were acquired with a pixel size between 0.3 and 1.0 nm—depending on the bubble size. The matrix background in Fig. 1c was taken from a 10 × 10 pixel window next to the bubble and was then subtracted from the total measured spectrum. The residual signal was then integrated between 10–15 eV and 21–25 eV to map the spatial H and He distribution shown in Fig. 2, respectively. The spectra in Fig. 1d were normalized to the base level between 105–110 eV. The pixel sizes for the SIs in Fig. 2 are 1 nm, the exposure time per pixel was set to 0.5 s and the obtained energy resolution was 0.18 eV. For the time series acquisition in Fig. 3 we acquired EELS spectra with an exposure time of 0.002 s over a time scale of 10 min and 38 s. As for the previous measurement we choose a pixel size of 1 nm for the spectra for the opened bubble.

The thickness of the lamellae and the bubble size was measured using EELS and the log-ratio technique. For these calculations we determined the mean free path using the approach from Iakoubovskii et al.^57^. We verified the accuracy of the EELS bubble size measurements by geometrically estimating the bubble size from the high-angular annular dark-filed (HAADF) and bright-field (BF) images.

For the opening of the bubbles, we acquired a time series were the electron beam was parked at the position indicated by cross (2) in Fig. 1a and consecutive EEL spectra were recorded. This procedure led to an opening of the bubble and a subsequent, abrupt release of He after 7 min and 45 s. Figure 3a shows the low-loss spectra before and after bubble opening from the same region for a 5 × 5 pixel window.

For the in situ experiments we used a DENSsolutions Wildfire^TM^ D6 double tilt holder with the corresponding MEMS nano heater chips. The four-point probe setup of this heater allows for a precise control of the temperature^58^. To keep the impact of the electron beam during the heating experiments as low as possible we used exposure and pixel times of and 0.00058 s and 0.01 s, respectively. To perform the step wise heating series we consecutively increased the temperature with a 10 min hold at each step before acquiring a SI. Temperature steps were reached instantaneously. We started all our measurements at 200 °C since preliminary investigations showed no effects below this temperature all spectra in Fig. 4 were taken from a 2 × 10 pixel window close to the bubble surface (see Supplementary Fig. 1) and scaled to their respective Be-bulk plasmon height at room temperature.

We performed our EDX measurements with an FEI Talos microscope equipped with an x-FEG and a windowless Super-X detector system which allows for high count rates and detecting low elemental concentrations. For data acquisition and evaluation, we used the Thermo Fisher Scientific (former FEI) Velox software package.

### Multivariate statistical analysis

We performed all our EELS data processing using Digital Micrograph from Gatan and the open source python data analysis toolbox hyperspy^59^ which can directly read Gatan standard.dm3 and.dm4 formats. After SI acquisition we used a weighted principal component analysis (wPCA) adapted to the Poissonian noise of the EELS data using the singular value decomposition algorithm in order to reduce the noise in our data.

### Density functional theory (DFT) calculations

In this work we performed DFT calculations revealing interaction of various impurities with free beryllium surfaces parallel to the crystallographic planes (0001) representing the largest surface areas inside facetted helium bubbles as illustrated in Fig. 1b.

For this purpose, slabs with the dimensions 4 × 4 × 12 were created for the basal (0001). Static ab initio calculations based on density functional theory were performed using the Vienna Ab initio Simulation Package (VASP)^60,61^. The generalized gradient approximation (GGA) of Perdew, Burke, and Ernzhof^62^ for the exchange-correlation energy was employed. The projector augmented wave pseudopotentials (PAW) for beryllium (2s^2^), aluminum (3s^2^ 3p^1^), iron (3d^6^ 4s^2^), magnesium (3s^2^), silicon (3s^2^ 3p^2^), helium (1s^2^) were taken from the VASP library of the PAW-GGA pseudopotentials^63,64^.

All calculations were performed with an electronic convergence criterion of EDIFF = 10^−5^ and a force-based ionic convergence criterion of EDIFFG = −0.005 eV/Å. A Fermi smearing of 0.2 eV and a plane wave cut-off energy of 486.814 eV were used. For sampling the Brillouin-zone, the KSPACING parameter is chosen to be equal to 0.12157. The choice of this parameter set is based on thorough ab initio calculations testing convergence of the results with respect to every parameter used and justified by a small entropy term, which is in this case less than 1.0 meV per atom.

During static simulations, both, volume and shape of the computational cell are fixed. Periodic boundary conditions along all crystallographic directions are applied, as implemented in VASP. The vacuum gap of the size of 37.10 Å was used for basal and prismatic type I surfaces, respectively, which was found to be sufficient to avoid interaction between two free surfaces arising due to the use of periodic boundary conditions.

The equilibrium lattice parameters for hcp lattice of pure beryllium at zero Kelvin and zero external pressure were found to be *a* = 2.267 Å and *c* = 3.561 Å, which are consistent with the previous publications^65–67^. All atomic layers were allowed to relax, i.e. no fixed atomic positions.

A single impurity atom was placed at representative positions at different distances from the surfaces. In order to characterize how these impurity atoms are bound to the surfaces, the energy difference between the configurations, where the impurity atom is located at the largest possible distance from the free surface (which energy is very close to the energy of impurity in the bulk of material) and at some distance from the surface, is computed

$${E}_{{{{{{\rm{gain}}}}}}}={E}_{{{{{{\rm{bulk}}}}}}}^{{{{{{\rm{Be}}}}}}+X}-{E}_{n}^{{{{{{\rm{Be}}}}}}+X}.$$

Here ${E}_{{{{{{\rm{bulk}}}}}}}^{{{{{{\rm{Be}}}}}}+X}$ is the energy of the computational cell, where impurity atom *X* (*X* = Al, Fe, Mg, Si) is located in the middle of the slab; ${E}_{n}^{{{{{{\rm{Be}}}}}}+X}$ is the energy of the cell where impurity atom *X* is in the *n-th* crystal plane parallel to the free surface (where *n* = 1, 2, 3, 4) below the free surface.

## Supplementary information

Supplementary Information

## Data Availability

The data that support the findings of this study are available from the corresponding author upon reasonable request.
