# Supplementary material for: Hydrogen and helium trapping in hcp beryllium
Source: Commun Chem. 2023 Apr 21;6:76. doi: 10.1038/s42004-023-00877-7 (PMC10121688; doi:10.1038/s42004-023-00877-7)
Supplement: Supplementary file 1 — Supplementary Information [file 42004_2023_877_MOESM1_ESM.pdf]

Supplementary Methods

Measured bubbles for the in situ heating experiment

For the heating experiments shown in the main part of the publication, we used the bubbles shown in Supplemetary Figure 1. Bubbles were located next to each other on the same lamella. The spectra presented in Figure 4 are taken from the yellow areas at the respective bubble surfaces.

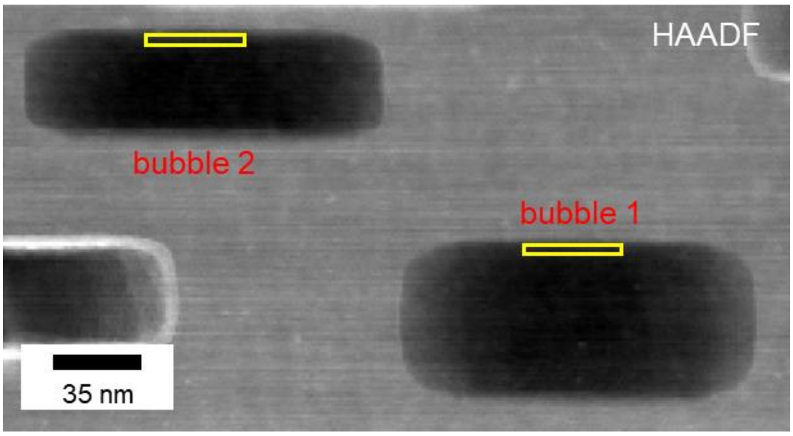

Supplementary Figure 1: Bubbles used for the in situ heating comparison shown in the main article. Bubble one was cut open with the electron beam before heating. The yellow rectangles mark the areas for the spectra shown in Figure 4. [1]

BeO

As mentioned in the main part of the article the plasmon at around 8-9 eV can be attributed to the formation of BeO at the surface of the lamellae. Changes in the Be K-edge can only be observed if the sample is reasonably thin ≤ 40 nm so that the ratio of BeO/Be is high. Otherwise the matrix contribution of non-oxidized Be to the K-edge is too big.

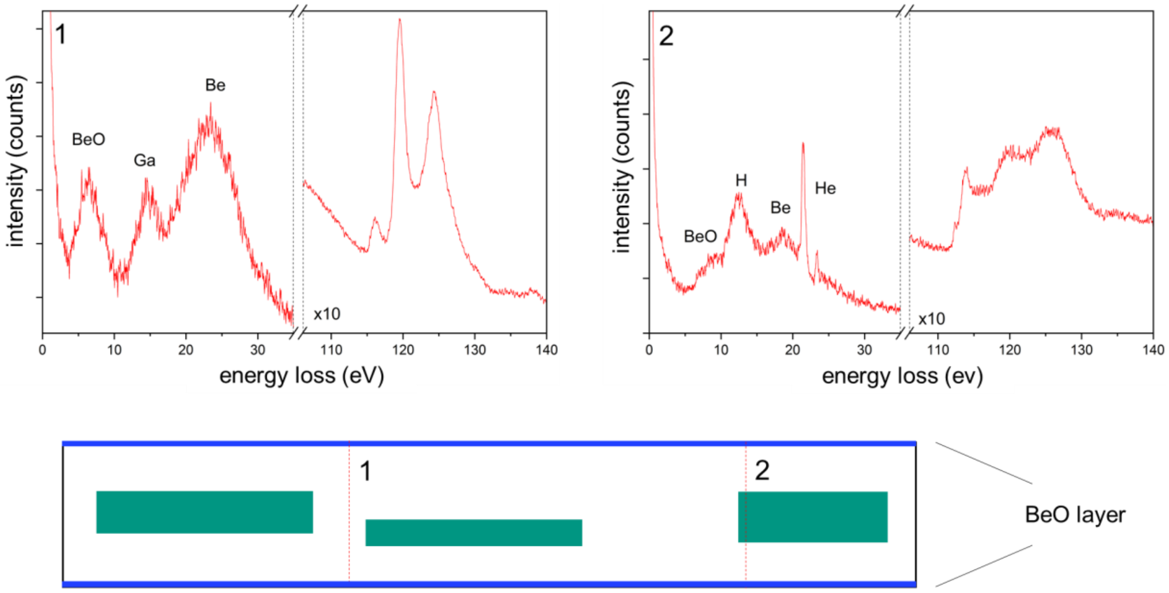

Supplementary Figure 2: Change of the Be K-edge with oxidation state: (1) strongly oxidized, (2) weakly oxidized. The sample must be reasonably thin to observe this change in the edge shape. The spectra show the change of the Be K-edge due to the formation of BeO which is good agreement with earlier investigations [1–3].

Bubbles at elevated temperatures

If bubbles are not opened during the heating procedure, both helium and hydrogen intensities do not change significantly at elevated temperatures.

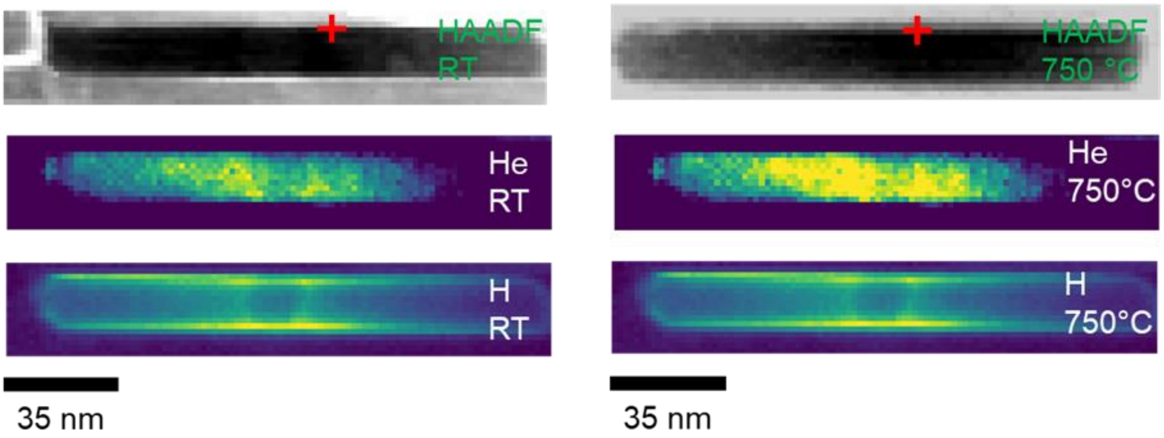

Supplementary Figure 3: He and H in a closed gas bubble at room temperature (RT) and at 750 °C. [1] The red crosses mark the position for the spectra shown in A 4.

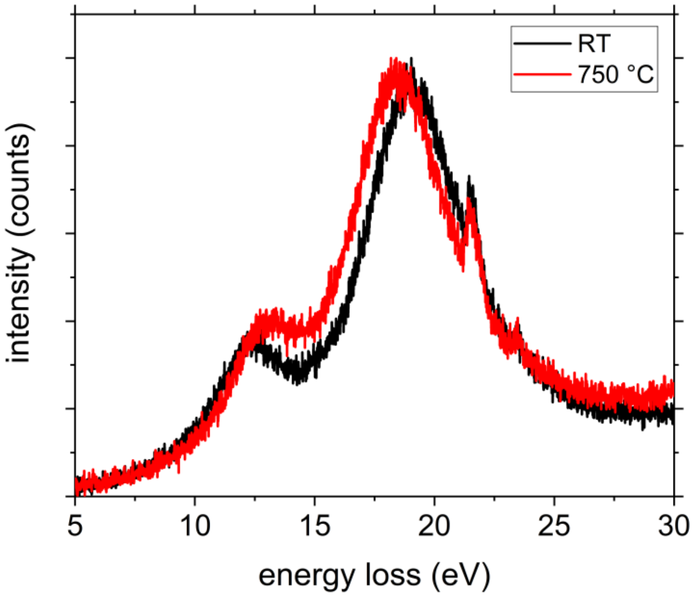

Supplementary Figure 4: EELS signal from the bubble surface of the bubble shown in A 3 (see red crosses) at room temperature (RT) and 750 °C. The increased temperature leads to a decrease in the valance electron density which is visible as a Be bulk plasmon shift to the left. [4]

Helium density, pressure determination and helium signal

A common procedure to estimate the He density in closed gas bubbles is the one presented by Walsh et al. [5] The He density n_{He} is given by

$$n_{\mathrm{He}} = \frac{I_{\mathrm{He}}}{I_{\mathrm{ZLP}}} \frac{1}{d\sigma_{\mathrm{He}}}, \tag{1}$$

where I_{He} and I_{ZLP} are the integrated intensities of the He K-edge and the zeroloss-peak, respectively d is the bubble thickness at the pixel position and σ_{He} the cross-section of the He 1s→2p transition, which was calculated using Sigmak3 [4]. The error of this density calculation is about ± 30 % and is mainly caused by the thickness measurements. With an acceleration voltage of 300 kV and a semi collection semi-angle of 18.8 mrad our

experimental setup leads to a cross-section of the He 1s→2p transition of 7.747 × 10^{-19} cm^2 for a 4 eV integration window.

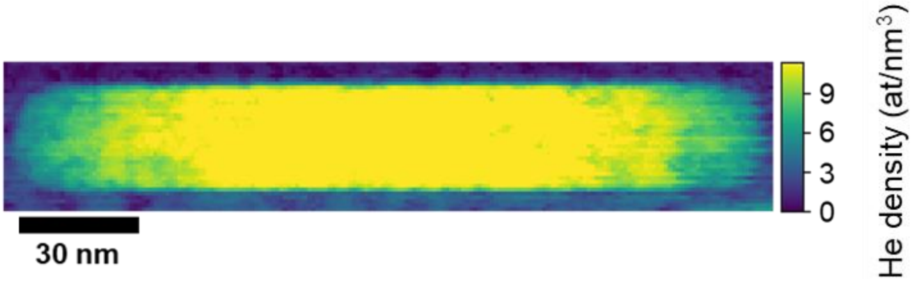

Supplementary Figure 5: Helium density inside a closed gas bubble in Be using equation (1). [1]

The pressure inside the bubble can be estimated using the semi-empirical equation of states (EOS) proposed by Trinkaus [6]. The pressure P inside a bubble is given as a function of the helium density n in atoms/angstroms and the temperature T in Kelvin.

$$P = 0.0138062nT\left[(1-vn)(1+vn-2v^2n^2) + n\left(\frac{170}{T^{\frac{1}{3}}} - \frac{1750}{T}\right)(1-vn)^2 + 0.1225(3-2vn)v^3n^2T^{0.555} - 50(1-vn)v^2n^2\right] \quad (2)$$

The coefficient v is given by

$$v = \frac{56}{T^{1/4}} \exp -\left(0.145T^{\frac{1}{4}}\right). \quad (3)$$

For the bubble in A 5, this results in a pressure of 4.4 × 10^{-2} GPa at 293 K.

Supplementary Figure 6 shows the helium blue-shift due to the high helium density in the bubble interior.

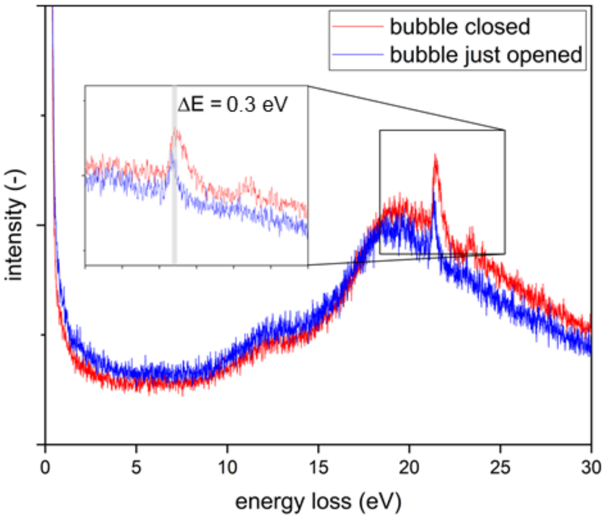

Supplementary Figure 6: Helium peak shift during bubble opening. The red curve shows the signal of a closed bubble. The blue signal was acquired just after the bubble was opened and the pressure dropped consequently. [1]

Furthermore - for the first time - we measured a high-energy-resolution EEL spectrum of helium that shows four different electron transitions (see Figure 1 (c)) using STEM EELS. In

the past only the 1s→2p and 1s→3p transitions have been measured using EELS [7–10]. More transitions were only visible in specially designed electron scattering experiments [11].

Hydrogen peak

Theoretically the observed feature at around 12–13 eV at the bubble walls could be attributed to the Be-surface plasmon. According to the Drude Model the position of the surface plasmon E_s in the spectrum is given by

$$E_S = \frac{E_P}{\left(\frac{2l+1}{l+1}\right)^{\frac{1}{2}}}, \tag{4}$$

where E_p is the bulk plasmon energy. The surface plasmon would appear in the range of $E_p/\sqrt{3/2}$ for l=1 and $E_p/\sqrt{2}$ for $l\rightarrow\infty$ which gives 13.2–15.3 eV. However, in our opinion several things speak against the surface plasmon and for hydrogen:

(i) Not all closed bubbles, even if they had the same sizes, showed the 12–13 eV peak.

(ii) The 12-13 eV peak showed similar intensities for variously sized bubbles.

(iii) Bubbles that have been cut open already during FIB preparation of the lamellae do not show the 12–13 eV peak. It is known, that Beryllium oxides very quickly, however the formation of thin oxide-layers does not necessarily prevent the occurrence of a surface plasmon. [12]

(iv) The 12–13 eV peak is also present in the bubble interior, albeit with a weaker intensity. The strong delocalisation of surface plasmons was only reported for energies > 3 eV in the past. Surface plasmons with energies < 10 eV undergo only a shift of several nanometers. [12]

(v) The observed peak at 12-13 eV was located constantly at the same energy within the individual bubbles and was generally slightly below the expected energy range for the beryllium surface plasmon.

(vi) For us there is no reason why the plasmon peak intensity should decrease with increasing temperature as strong as we observed it. Although there have been reports about a red shift and broadening of the surface plasmon resonance in Au nanoparticles as the result of an increased temperature [13], they do not disappear completely as it is the case in our experimental investigations.

(vii) Recent Atom Probe Tomography examinations at the Culham Science Center for Fusion Energy (CCFE) [14] detected appearance of the tritium related mass-spectrum peaks in the same neutron irradiated beryllium samples used in this work. The corresponding detected atoms appeared in disk-like shapes with dimensions similar to the helium-tritium bubbles observed in the present EELS work. The APT data is still under evaluation and will be soon published in a separate research paper.

(viii) Temperature Programmed Desorption (TPD) experiments [15–18] revealed simultaneous “burst” release of helium and hydrogen from bulk samples when irradiated Be pebbles are heated to temperatures ≤ 1100 °C. This observation indicates that both elements, helium and hydrogen, must be present within bubbles.

(ix) DFT simulations revealed that hydrogen atoms which adsorb on Be (0001) surfaces may lead to a substantial reconstruction of the beryllium surface [19,20]. Since hydrogen atoms prefer a two-fold coordinated bridge position BeH_2 polymer chains are formed. Polymer-like structures, however, will have a different electronic structure compared to the bulk material and hence also a deviating

EELS signal. As a consequence, the calculated energies from the Drude Model might not be correct in our case.

Supplementary References

[1] N. Zimber, Nanoskalige Analytik der Mikrostruktur von hochdosig bestrahltem Beryllium: PhD Thesis, DOI: 10.5445/IR/1000139959, Karlsruher Institute of Technology (KIT), 2021.

[2] M. Klimenkov, V. Chakin, A. Moeslang, R. Rolli, TEM study of beryllium pebbles after neutron irradiation up to 3000appm helium production, Journal of Nuclear Materials 443 (2013) 409–416. https://doi.org/10.1016/j.jnucmat.2013.07.050.

[3] C. Makepeace, C. Pardanaud, P. Roubin, I. Borodkina, C. Ayres, P. Coad, A. Baron-Wiechec, I. Jepu, K. Heinola, A. Widdowson, S. Lozano-Perez, J.E.T. Contributors, The effect of beryllium oxide on retention in JET ITER-like wall tiles, Nuclear Materials and Energy 19 (2019) 346–351. https://doi.org/10.1016/j.nme.2019.02.022.

[4] R.F. Egerton, Electron Energy-Loss Spectroscopy in the Electron Microscope, 3rd ed., Springer Science+Business Media LLC, Boston, MA, 2011.

[5] C.A. Walsh, J. Yuan, L.M. Brown, A procedure for measuring the helium density and pressure in nanometre-sized bubbles in irradiated materials using electron-energy-loss spectroscopy, Philosophical Magazine A 80 (2000) 1507–1543. https://doi.org/10.1080/01418610008212134.

[6] H. Trinkaus, Energetics and formation kinetics of helium bubbles in metals, Radiation Effects 78 (1983) 189–211. https://doi.org/10.1080/00337578308207371.

[7] K. Alix, M.-L. David, G. Lucas, D.T.L. Alexander, F. Pailloux, C. Hébert, L. Pizzagalli, Gentle quantitative measurement of helium density in nanobubbles in silicon by spectrum imaging, Micron (Oxford, England 1993) 77 (2015) 57–65. https://doi.org/10.1016/j.micron.2015.05.011.

[8] R. Schierholz, B. Lacroix, V. Godinho, J. Caballero-Hernández, M. Duchamp, A. Fernández, STEM-EELS analysis reveals stable high-density He in nanopores of amorphous silicon coatings deposited by magnetron sputtering, Nanotechnology 26 (2015) 75703. https://doi.org/10.1088/0957-4484/26/7/075703.

[9] GATAN, EELS Atlas, https://eels.info/atlas, accessed 25 August 2020.

[10] M. Klimenkov, P. Vladimirov, J. Hoffmann, N. Zimber, A. Möslang, V. Kuksenko, First simultaneous detection of helium and tritium inside bubbles in beryllium, Micron (Oxford, England 1993) 127 (2019) 102754. https://doi.org/10.1016/j.micron.2019.102754.

[11] Khakoo, Roundy, Rugamas, Electron-impact excitation of the 1 1S--3 1P and 1 1S--4 1P transitions in helium, Physical review. A, Atomic, molecular, and optical physics 54 (1996) 4004–4014. https://doi.org/10.1103/PhysRevA.54.4004.

[12] C. Colliex, M. Kociak, O. Stéphan, Electron Energy Loss Spectroscopy imaging of surface plasmons at the nanometer scale, Ultramicroscopy 162 (2016) A1-A24. https://doi.org/10.1016/j.ultramic.2015.11.012.

[13] O.A. Yeshchenko, I.S. Bondarchuk, V.S. Gurin, I.M. Dmitruk, A.V. Kotko, Temperature dependence of the surface plasmon resonance in gold nanoparticles, Surface Science 608 (2013) 275–281. https://doi.org/10.1016/j.susc.2012.10.019.

[14] V. Kuksenko, Personal Correspondence, Culham Centre for Fusion Energy, March 2021.

[15] V. Chakin, R. Rolli, A. Moeslang, M. Klimenkov, M. Kolb, P. Vladimirov, P. Kurinskiy, H.-C. Schneider, S. van Til, A.J. Magielsen, M. Zmitko, Tritium release and retention properties of highly neutron-irradiated beryllium pebbles from HIDOBE-01 experiment, Journal of Nuclear Materials 442 (2013) S483-S489. https://doi.org/10.1016/j.jnucmat.2013.03.032.

[16] P.V. Vladimirov, V.P. Chakin, M. Dürrschnabel, R. Gaisin, A. Goraieb, F.A.H. Gonzalez, M. Klimenkov, M. Rieth, R. Rolli, N. Zimber, S. Udartsev, M. Kolmakov, A. Vechkutov,

E. Frants, Development and characterization of advanced neutron multiplier materials, Journal of Nuclear Materials 543 (2021) 152593. https://doi.org/10.1016/j.jnucmat.2020.152593.
[17] V. Chakin, R. Rolli, M. Klimenkov, M. Zmitko, Tritium release and retention in beryllium pebbles irradiated up to 640 appm tritium and 6000 appm helium, Journal of Nuclear Materials 542 (2020) 152521. https://doi.org/10.1016/j.jnucmat.2020.152521.
[18] V. Chakin, R. Rolli, R. Gaisin, U. Hoeppener-Kramar, M. Nakamichi, M. Zmitko, Tritium release and retention in beryllium and titanium beryllide after neutron irradiation up to damage doses of 23-38 dpa, Fusion Engineering and Design 161 (2020) 111938. https://doi.org/10.1016/j.fusengdes.2020.111938.
[19] A. Allouche, Density functional study of hydrogen adsorption on beryllium (0001), Phys. Rev. B 78 (2008). https://doi.org/10.1103/PhysRevB.78.085429.
[20] E.N. Koukaras, A.P. Sgouros, M.M. Sigalas, Fully Hydrogenated Beryllium Nanoclusters, Journal of the American Chemical Society 138 (2016) 3218–3227. https://doi.org/10.1021/jacs.6b00135.
